# Evaluating Patient Outcomes and Access to Care in Aortic Surgery Based on Ethnicity and Social Vulnerability

**DOI:** 10.1055/a-2608-1346

**Published:** 2025-06-03

**Authors:** Ananya Shah, Adam M. Carroll, Nicolas Chanes, Kyndall Hadley, Cenea Kemp, Bo Chang Brian Wu, Alejandro Suarez-Pierre, Jessica Rove, Catherine Velopulos, Muhammad Aftab, T. Brett Reece

**Affiliations:** 1 Department of Surgery, University of Colorado School of Medicine, Aurora, Colorado; 2 Division of Cardiothoracic Surgery, Department of Surgery, University of Colorado School of Medicine, Aurora, Colorado

**Keywords:** aortic arch surgery, social vulnerability index, health disparities, ethnicity and surgical outcomes, access to care

## Abstract

**Background:**

We previously demonstrated the impact of ethnicity on aortic surgery, with underrepresentation and greater acuity in minority patients, raising concerns regarding access to care. The Centers for Disease Control and Prevention's social vulnerability index (SVI) measure is increasingly used to quantify patient socioeconomic and demographic factors. This study expands on our prior work by incorporating SVI and ethnicity to analyze patient presentation and outcomes in aortic arch surgery.

**Methods:**

We utilized a single-institution database of patients who underwent total arch replacement or hemiarch repair between 2009 and 2022. A total of 837 patients were placed into five cohorts based on their self-reported race: African American, Asian, Caucasian, Hispanic, and Other, with further subdivision based on SVI (high social vulnerability, ≥75%, normal social vulnerability < 75%). Additional analyses were performed using SVI alone. We compared patient presentation, operative variables, and outcomes based on the above cohorts.

**Results:**

African American and Hispanic patients were underrepresented compared with city demographics. High SVI and minority patients presented at younger ages ( *p* = 0.007) with higher blood pressures ( *p* = 0.002). These groups also had more urgent/emergent presentations ( *p* < 0.001) with aortic dissections ( *p* = 0.006). Operatively, high SVI groups had longer cardiopulmonary bypass ( *p* = 0.018), cross-clamp ( *p* = 0.020), and circulatory arrest times ( *p* = 0.002) but fewer adjunctive procedures ( *p* = 0.018). High SVI patients more often required total arch replacement ( *p* = 0.048) and postoperative mechanical circulatory support ( *p* = 0.025). After discharge, African Americans had more emergency department (ED) visits within a year ( *p* < 0.001), although no significant differences were observed in readmission rates or cardiovascular follow-up.

**Conclusion:**

Underrepresented groups face barriers to care, as reflected in disparities in demographics, surgical acuity, and postdischarge ED usage. Analyses-based solely on ethnicity overlooked critical differences between normal and high SVI groups, emphasizing the need for care strategies that are both tailored to high SVI groups and racially sensitive applied across all levels of health care.

## Introduction

There have been several studies demonstrating the influence that ethnicity and social vulnerability have on disease severity, morbidity, and mortality. 1 2 3 4 Such data highlight the importance of equitable access to care and underscores the need for continued research to help mitigate disparities. While the individual impact of different demographic and socioeconomic factors has been studied, few have investigated the combined effects of the intersecting variables. Analyzing the cumulative impact of these factors may provide insight into how social vulnerability affects different ethnicities and provide a more targeted approach to improving access to care.

The CDC's social vulnerability index (SVI) quantifies neighborhood-level disparity, with a higher SVI indicating more social vulnerability. This measure has been used to study the impact of social vulnerability on surgical outcomes in trauma, coronary artery disease, and abdominal aortic aneurysms; however, it has not been applied to aortic arch surgery. 5 6 7 8 Given the heterogeneity across racial groups and socioeconomic classes, it is of paramount importance to evaluate their impact in aortic surgery.

The purpose of this study is to investigate differences in presentation, outcomes, and follow-up across different racial and socioeconomic groups in aortic arch surgery. Furthermore, performing simultaneous analysis of race and the individual and cumulative effects of SVI will better define presentation and subsequent outcomes. The results of this study will identify and help develop tailored approaches to expand equitable care for aortic surgery patients.

## Materials and Methods

A retrospective review of a single institution prospectively maintained aortic database was performed for all patients who underwent aortic arch surgery from 2011 to 2022. All adults (age 18 years or older) who underwent open aortic arch surgery, including hemiarch, total arch, or other aortic arch replacement were included. In total, 837 patients were identified. All data were collected through manual review of these medical records in compliance with policies of the institution. The study design was reviewed and approved for exception by the Colorado Multiple Institutional Review Board (COMIRB #17-0198, approval date: February 6, 2017).

Data compiled within this registry include data submitted to the Society of Thoracic Surgeons (STS) as well as other unique variables, including demographic characteristics as well as preoperative, intraoperative, and outcome variables. In total, 92 variables were included in the analyses. Preoperative variables, including age, sex, body mass index, history of medical comorbidities such as hyperlipidemia, hypertension, type 2 diabetes mellitus, chronic kidney disease, pulmonary disease, coronary artery disease, and smoking history were included. Ethnicity was obtained from the medical record as self-reported by patients. Additionally, baseline variables including systolic and diastolic blood pressures, international normalized ratio (INR), creatinine (Cr), and hemoglobin A1C were obtained. For elective patients, INR, Cr, and blood pressures were obtained from preoperative clinic visits or laboratory appointments. For nonelective patients, these values were taken from either primary care or other provider visits within 3 months of surgery. If these variables were not obtainable within 3 months prior to presentation, these values were obtained from vitals or laboratories at the time of initial presentation if there was no evidence of malperfusion, shock, or hypertensive crisis. In the few nonelective cases where patients did not have any prior clinical assessment within 3 months of operation and presented in any of the above conditions, these values were excluded from analysis. Baseline hemoglobin A1c was obtained either from preoperative assessments or from initial postoperative laboratories (within first 24 hours of surgery).

For operative and postoperative variables, these were obtained through manual review of operative and medical records. Regarding postoperative in-hospital morbidity and mortality, postoperative stroke was defined as new-onset neurological deficit lasting >24 hours with imaging evidence of infarction or hemorrhage. Operative mortality was based off the STS definition and included all deaths occurring within the index hospital stay or death within 30 days of the procedure if discharged. New renal replacement therapy requirement included acute kidney injury requiring hemodialysis.

Additionally, patient follow-up with a cardiovascular provider postdischarge was assessed, along with a composite of cardiovascular- or procedure-related emergency department (ED) visits as well as unplanned readmissions within one year of surgery. In addition to reviewing patient-provided information, CareEverywhere was also searched for these variables to ensure thorough data collection. Cardiovascular-related presentations were defined as any presentation related to concern for arrhythmia, myocardial infarction, heart failure, new-onset renal failure, hypertensive crisis, or thromboembolic event. Procedure-related presentations included postoperative infections, pain associated with the operation or baseline aortic pathology, hemorrhagic events related to postprocedure anticoagulation, or unplanned readmissions for the management of aortic pathology.

### Social Vulnerability Index Assessment

This study implemented custom built Python code (version 3.12.2, Python Software Foundation) to develop an automated system for SVI calculation that replicates the manual process available via the interactive CDC SVI map. The system utilized the 2020 SVI census tract dataset in conjunction with geocoded address data from the patient population to evaluate social vulnerability. 9 Address data were matched to SVI data using Federal Information Processing Standards (FIPS) codes, which were derived from geospatial longitude and latitude coordinates. Custom data processing techniques were employed to align geocoded patient address data with the validated CDC SVI dataset. These techniques included the use of regular expressions for sophisticated text data manipulation and numerical transformation methods to ensure accurate data alignment. Regular expressions are a powerful tool used for pattern matching and manipulation within text, enabling sophisticated search and replace operations in strings of data. Geocoding was conducted using the U.S. Census Bureau geocoder tool, which relies on the Master Address File/Topologically Integrated Geographic Encoding and Referencing (MAF/TIGER) database to convert addresses or location coordinates into geocoded information. Designed to facilitate efficient and precise public geocoding, this tool provides results based on an address match score. This geocoding tool yields interpolated coordinates by approximating the physical location of an address within the TIGER database address ranges. FIPS codes were generated in accordance with the National Institute of Standards and Technology (NIST) to precisely match geocoded addresses with their relevant SVI metrics. The analysis identified the SVI percentile ranking for each address, highlighting those ranked in the 75th percentile or higher. This categorization process was designed to identify areas of heightened vulnerability, thereby facilitating the analysis of potential correlations between social determinants and health outcomes.

### Analysis

Subgroup analyses were undertaken to elucidate the associations of ethnicity, SVI, and their combination with various clinical and demographic outcomes. Continuous variables were subjected to inferential statistics including analysis of variance for ethnicity and combined ethnicity SVI groups, and the Mann–Whitney U test (also known as the Wilcoxon rank-sum test) for differentiating SVI subgroups. Binary variables underwent hypothesis testing with Fisher's exact tests across all aforementioned groups, establishing a significance level at a *p* -value of ≤0.05.

Comparative assessments were performed on a range of demographic and clinical variables, including operation urgency, history of aortic dissection, tobacco smoking, and incidence of total arch replacements. This involved contrasting Caucasian against African American subgroups, as well as high SVI (≥75%) against low SVI (<75%) categories. Furthermore, a cross-analysis considering both ethnicity and SVI compared Caucasian patients with low SVI to their African American counterparts with high SVI values. Contingency tables were constructed to calculate odds ratios and *p* -values via the Fisher's exact test. Odds ratios were accompanied by 95% confidence intervals computed using the standard error of the log of the odds ratio. To present these associations visually, a forest plot was generated, highlighting the effect sizes and corresponding confidence intervals for each comparison.

## Results

Regarding racial and ethnic distribution of the cohort, 76.6% (641/837) identified as Caucasian, 9.7% (81/837) identified as Black, 8.7% (73/837) identified as Hispanic, 2.4% (20/837) identified as Asian, and 2.6% (22/837) identified as Other. Demographic city data compared with included patients were 16.6 versus 9.7% for the African American cohort, 6.6 versus 2.4% for the Asian cohort, 43.5 versus 76% for the Caucasian cohort, 12 versus 8.7% for the Hispanic cohort, and 22 versus 2.6% for the Other cohort. The patient cohort was not consistent with city demographics, with under representation of non-Caucasian minorities in the patient cohort.

**Fig. 1:**
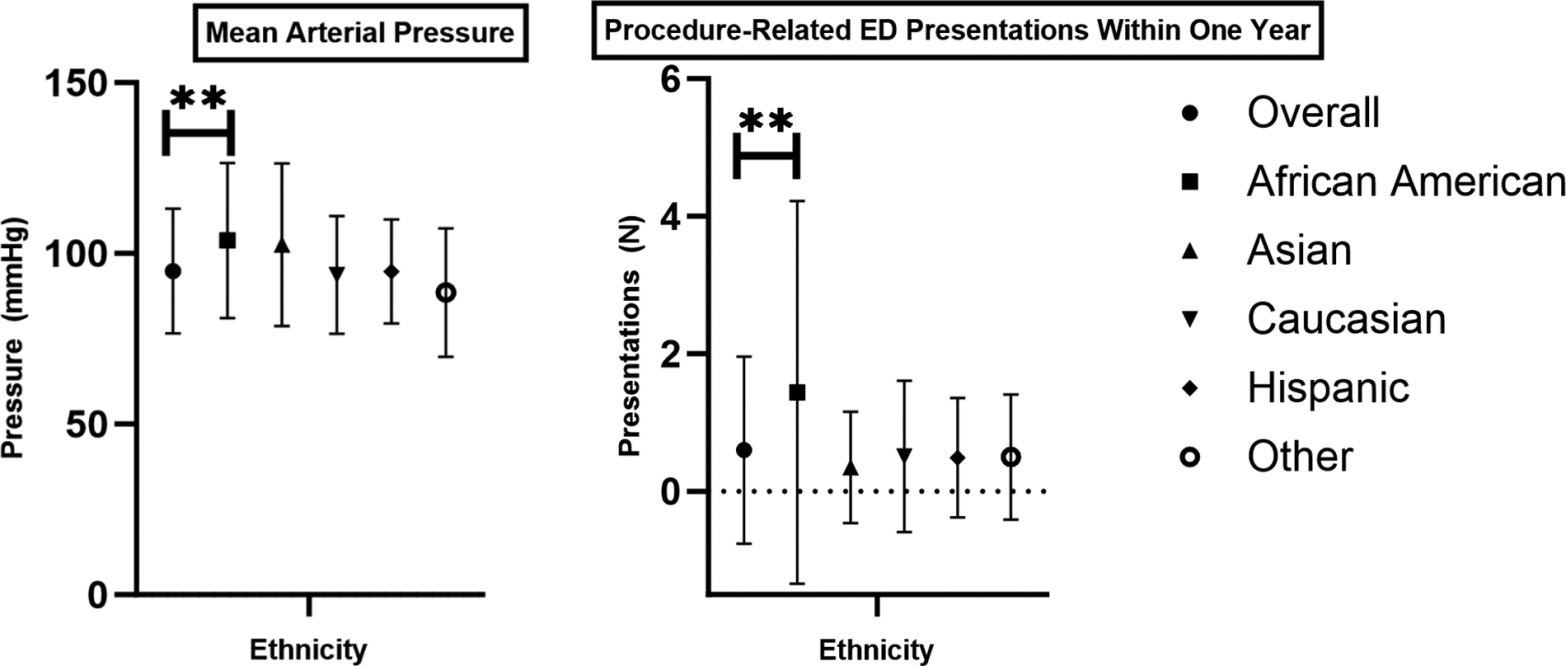
Comparative analysis of mean arterial pressure (MAP) and procedure-related emergency department (ED) presentations within 1 year, categorized by ethnicity.

As seen in Table 1 , Regarding ethnicity only, African American and Hispanic individuals presented at a younger age ( *p* = 0.001). African American patients also presented with higher baseline systolic ( *p* = 0.002) and diastolic blood pressures ( *p* < 0.001) and were significantly more likely to present urgently or emergently ( *p* = 0.002) with aortic dissection pathology ( *p* = 0.003). Regarding intraoperative characteristics, African Americans and Asians required longer cardiopulmonary bypass (CPB; *p* = 0.029), aortic cross-clamp ( *p* = 0.012), and circulatory arrest times ( *p* = 0.005). Postoperatively, African Americans were more likely to have procedure-related ED presentations within 1 year of surgery ( *p* < 0.001) as seen in Fig. 1 . However, no statistically significant differences were seen for length of stay, in-hospital morbidity, in-hospital mortality, readmission, or follow-up rates with cardiovascular providers when investigating ethnicity alone.

As seen in Table 2 , Regarding the SVI analysis alone, SVI was categorized as <75% or SVI ≥ 75%. Preoperatively, high SVI patients presented at a younger age ( *p* = 0.007), had a history of smoking ( *p* = 0.007), and presented urgently or emergently ( *p* = 0.001) as compared with lower SVI patients. There were no differences in BMI, baseline laboratory data, aortic presentation, past cardiothoracic or aortic surgical history, or other comorbidities. Intraoperatively, patients with high SVI were more likely to require total arch replacement ( *p* = 0.048) and have significantly longer CPB times ( *p* = 0.008), aortic cross clamp times ( *p* = 0.048), and circulatory arrest times ( *p* = 0.001). They also had a lower intraoperative nadir bladder temperature ( *p* < 0.001). There was no significant difference in the amount of intraoperative blood product transfusion. Postoperatively, high SVI patients required more mechanical circulatory support ( *p* = 0.025) and utilized the ED more frequently in the first year after surgery ( *p* = 0.003). There were no significant differences in length of stay, intensive care unit morbidity, follow-up rates, or mortality for patients in the higher SVI group.

**Fig. 2:**
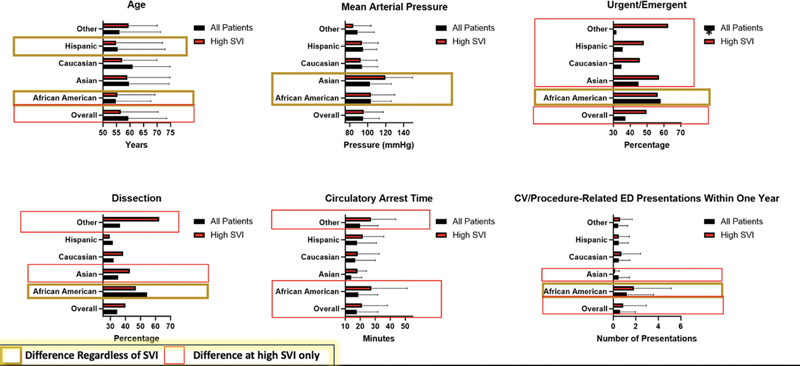
Cumulative impact of SVI and ethnicity on preoperative, intraoperative, and postoperative characteristics. SVI, socioeconomic vulnerability index.

**Fig. 3:**
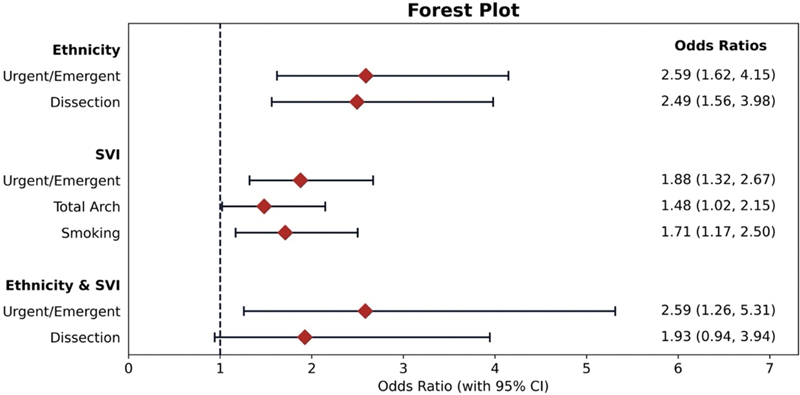
Forest plot depicting the odds ratios (OR) with 95% confidence intervals (CI) for various risk factors, stratified by ethnicity, socioeconomic vulnerability index (SVI), and their combination. The Ethnicity comparison demonstrates the differences in risk between African American versus Caucasian patients for urgent/emergent procedures and dissections. The SVI comparison depicts the differences between SVI ≥ 75 versus SVI < 75 patients for urgent/emergent procedures, total arch surgeries, and smoking status. The combination of ethnicity and SVI comparison demonstrates the differences between African American patients with SVI ≥ 75 versus Caucasian patients with SVI < 75 for urgent/emergent procedures and dissections.

As seen in Fig. 2 , several of the differences between ethnicities became statistically significant when the cumulative impact of SVI and ethnicity was considered. Preoperatively, regardless of SVI, African American and Hispanic patients presented at a younger age ( *p* = 0.001), with high SVI patients in general more likely to present at a younger age ( *p* = 0.007). African American and high SVI Asian patients presented with higher baseline systolic and diastolic blood pressures ( *p* = 0.002). African American and high SVI patients, regardless of race, were significantly more likely to present urgently or emergently ( *p* < 0.001) with aortic dissection pathology ( *p* = 0.006) as seen in Fig. 3 .

Intraoperatively, African American individuals had longer circulatory arrest times than other ethnicities only at high SVI ( *p* = 0.002). Regardless of ethnicity, patients with high SVI were more likely to require total arch replacement ( *p* = 0.048) and have significantly longer CPB times ( *p* = 0.008), aortic cross clamp times ( *p* = 0.048), and circulatory arrest times ( *p* = 0.001). There were no differences observed between the groups in the use of procedures involving stent grafts, such as frozen elephant trunks, nor were there disparities associated with ethnicity or SVI in the application of techniques utilizing advanced devices.

Postoperatively, significant differences were seen in the number of procedure-related ED presentations within 1 year ( *p* < 0.001), with notably high usage among African Americans regardless of SVI and low usage among high SVI Asian patients. No significant differences were seen in rates of readmission or follow-up with a cardiovascular provider.

In the multivariate logistic regression analysis, neither race nor SVI ≥ 75% were independently associated with postoperative mortality or readmission following aortic arch surgery. Urgent or emergent operative status emerged as a significant predictor of increased postoperative mortality (odds ratio [OR]: 3.16, 95% confidence interval [CI]: 1.44–6.93, *p* = 0.004), emphasizing the critical role of surgical acuity in patient outcomes ( Table 3 ). Other intraoperative factors, including CPB time (OR: 1.02, 95% CI: 1.01–1.03, *p* < 0.001) and nadir bladder temperature (OR: 1.22, 95% CI: 1.06–1.41, *p* = 0.006), were significantly associated with mortality risk, whereas cross-clamp time showed a mild protective effect (OR: 0.99, 95% CI: 0.98–1.00, *p* = 0.005). Variables such as BMI and comorbidities demonstrated limited or borderline significance, underscoring the multifactorial nature of outcomes in this patient population. These findings suggest that intraoperative management strategies and the urgency of surgical presentation are pivotal in mitigating mortality risk.

**Table 1:** Analysis of preoperative, intraoperative, and postoperative variables by race and ethnicity

Variable	Overall	African American	Asian	Caucasian	Hispanic	Other	*p* -Value
Sample size	837	81 (9.7%)	20 (2.4%)	641 (76.6%)	73 (8.7%)	22 (2.6%)	N/A
**Pre** o **perative** c **haracteristics**							
Age	61 [50, 69]	57 [45, 65]	61 [49, 73]	62 [52, 70]	57 [46, 67]	60 [46, 69]	0.002
Sex (male)	599 (71.6%)	65 (80.2%)	14 (70.0%)	455 (71.0%)	52 (71.2%)	13 (59.1%)	0.307
BMI	27.6 [24.3, 31.9]	28.0 [24.2, 32.3]	23.0 [20.6, 25.7]	27.6 [24.5, 31.9]	27.5 [24.3, 30.7]	26.7 [22.4, 33.3]	0.035
Baseline systolic BP	130 [117, 144]	140 [125, 156]	129 [121, 144]	129 [116, 142]	130 [118, 144]	130 [102, 147]	0.002
Baseline diastolic BP	75 [67, 86]	80 [70, 95]	80 [75, 85]	75 [67, 85]	74 [70, 86]	70 [58, 82]	<0.001
HTN	575 (68.7%)	67 (82.7%)	16 (80.0%)	429 (66.9%)	46 (63.0%)	17 (77.3%)	0.023
Smoking	203 (24.3%)	23 (28.4%)	6 (30.0%)	154 (24.0%)	17 (23.3%)	3 (13.6%)	0.641
Renal disease	88 (10.5%)	16 (19.8%)	3 (15.0%)	60 (9.4%)	4 (5.5%)	5 (22.7%)	0.007
Aortic presentation							
Aneurysm	619 (74.0%)	52 (64.2%)	16 (80.0%)	482 (75.2%)	54 (74.0%)	15 (68.2%)	0.260
Dissection	289 (34.5%)	44 (54.3%)	7 (35.0%)	207 (32.3%)	23 (31.5%)	8 (36.4%)	0.003
Operative urgency							
Elective	525 (62.7%)	34 (42.0%)	11 (55.0%)	418 (65.2%)	47 (64.4%)	15 (68.2%)	0.002
Urgent emergent	312 (37.3%)	47 (58.0%)	9 (45.0%)	223 (34.8%)	26 (35.6%)	7 (31.8%)	0.002
**Intrao** perative characteristics							
Procedure type							
Hemiarch	602 (71.9%)	52 (64.2%)	12 (60.0%)	473 (73.8%)	51 (69.9%)	14 (63.6%)	0.214
Total arch	235 (28.1%)	29 (35.8%)	8 (40.0%)	168 (26.2%)	22 (30.1%)	8 (36.4%)	0.214
Operative variables							
CPB time	156 [123, 211]	170 [129, 234]	158 [124, 189]	152 [121, 208]	162 [133, 210]	195 [146, 239]	0.029
Cross-clamp time	98 [71, 135]	111 [79, 162]	91 [63, 127]	98 [71, 133]	97 [66, 135]	110 [89, 171]	0.016
HCA time	13 [8, 23]	17 [10, 26]	15 [11, 18]	12 [8, 22]	16 [8, 27]	18 [13, 32]	0.005
Nadir bladder temperature	26.9 [25.0, 28.0]	26.1 [23.5, 27.7]	26.9 [25.5, 27.9]	27.1 [25.3, 28.0]	26.6 [24.0, 27.8]	26.0 [24.4, 28.0]	0.115
Nadir hemoglobin	8.5 [7.5, 10.0]	8.4 [7.5, 9.2]	7.8 [7.4, 8.5]	8.6 [7.5, 10.1]	8.4 [7.7, 10.4]	7.7 [7.3, 8.1]	0.015
**Posto** perative characteristics							
Length of stay	8.0 [6.0, 13.0]	9.0 [7.0, 14.0]	9.0 [8.0, 13.3]	8.0 [6.0, 13.0]	7.0 [6.0, 10.0]	9.0 [7.0, 12.0]	0.490
ICU length of stay	3.0 [2.0, 6.0]	3.0 [2.0, 6.0]	4.0 [3.0, 5.5]	3.0 [2.0, 6.0]	3.0 [1.3, 4.0]	4.0 [3.0, 6.5]	0.581
Postoperative mortality	63 (7.5%)	5 (6.2%)	2 (10.0%)	46 (7.2%)	7 (9.6%)	3 (13.6%)	0.712
Postoperative readmission	229 (27.4%)	32 (39.5%)	4 (20.0%)	166 (25.9%)	22 (30.1%)	5 (22.7%)	0.113
ED presentations in 1 y	0.0 [0.0, 1.0]	1.0 [0.0, 1.0]	0.0 [0.0, 0.0]	0.0 [0.0, 1.0]	0.0 [0.0, 1.0]	0.0 [0.0, 0.8]	<0.001
Follow-up CV	728 (87.0%)	73 (90.1%)	18 (90.0%)	555 (86.6%)	63 (86.3%)	19 (86.4%)	0.648
Postoperative late death	47 (5.6%)	3 (3.7%)	1 (5.0%)	39 (6.1%)	4 (5.5%)	0 (0.0%)	0.708

Abbreviations: BP, blood pressure; CPB, cardiopulmonary bypass; CV, cardiovascular; HCA, hypothermic circulatory arrest; HTN, hypertension; ICU, intensive care unit; N/A, not applicable.

Note: Values are *n* (%) or median (25–75% interquartile range).

**Table 2:** Analysis of preoperative, intraoperative, and postoperative variables by socioeconomic vulnerability index

Variable	Overall	SVI < 75%	SVI ≥ 75%	*p* -Value
Sample size	837	682 (81.5%)	155 (18.5%)	N/A
Preoperative characteristics				
Age	61 [50, 69]	62 [51, 70]	59 [47, 67]	0.007
BMI	27.6 [24.3, 31.9]	27.5 [24.3, 31.6]	28.0 [24.0, 32.4]	0.247
Baseline systolic BP	130 [117, 144]	130 [117, 143]	129 [114, 147]	0.670
Baseline diastolic BP	75 [67, 86]	75 [67, 86]	77 [67, 87]	0.425
Smoking	203 (24.3%)	152 (22.3%)	51 (32.9%)	0.007
Operative urgency				
Elective	525 (62.7%)	447 (65.5%)	78 (50.3%)	<0.001
Urgent emergent	312 (37.3%)	235 (34.5%)	77 (49.7%)	<0.001
Intraoperative characteristics				
Procedure type				
Hemiarch	602 (71.9%)	501 (73.5%)	101 (65.2%)	0.048
Total arch	235 (28.1%)	181 (26.5%)	54 (34.8%)	0.048
Adjunctive root procedure	307 (36.7%)	251 (36.8%)	56 (36.1%)	0.948
No adjunctive structural procedure	191 (22.8%)	144 (21.1%)	47 (30.3%)	0.018
Operative variables				
CPB time	156 [123, 211]	152 [121, 208]	178 [130, 232]	0.008
Cross-clamp time	98 [71, 135]	97 [71, 134]	103 [72, 143]	0.048
HCA time	13 [8, 23]	12 [8, 22]	16 [10, 26]	<0.001
Nadir bladder temperature	26.9 [25.0, 28.0]	27.1 [25.3, 28.0]	26.0 [23.8, 27.8]	<0.001
Nadir hemoglobin	8.5 [7.5, 10.0]	8.5 [7.6, 10.0]	8.40 [7.3, 9.8]	0.532
Postoperative characteristics				
Length of stay	8.0 [6.00, 13.0]	8.0 [6.0, 13.0]	9.0 [7.0, 14.0]	0.053
ICU length of stay	3.0 [2.0, 6.0]	3.0 [2.0, 6.0]	4.0 [2.0, 6.0]	0.115
New hemodynamic support	47 (5.6%)	32 (4.7%)	15 (9.7%)	0.025
Postoperative mortality	63 (7.5%)	48 (7.0%)	15 (9.7%)	0.339
Postoperative readmission	229 (27.4%)	185 (27.1%)	44 (28.4%)	0.575
ED presentations in 1 y	0.0 [0.0, 1.0]	0.0 [0.0, 1.0]	0.0 [0.0, 1.0]	0.003
Follow-up CV	728 (87.0%)	599 (87.8%)	129 (83.2%)	0.192
Postoperative late death	47 (5.6%)	37 (5.4%)	10 (6.5%)	0.758

Abbreviations: BP, blood pressure; CPB, cardiopulmonary bypass; CV, cardiovascular; HCA, hypothermic circulatory arrest; HTN, hypertension; ICU, intensive care unit.

Note: Values are *n* (%) or median (25–75% interquartile range).

**Table 3:** Multivariate logistic regression analysis evaluating predictors of postoperative mortality following aortic arch surgery

Variable	OR (95% CI)	*p* -Value
Patient characteristics		
Age	1.02 (1.00, 1.05)	0.057
BMI	0.94 (0.88, 0.99)	0.029
Baseline systolic BP	1.01 (0.99, 1.02)	0.517
Baseline diastolic BP	0.99 (0.97, 1.01)	0.445
SVI ≥ 75%	1.12 (0.49, 2.57)	0.794
Race		
African American	3.46 (0.45, 26.43)	0.232
Asian	3.04 (0.39, 23.64)	0.288
Caucasian	2.15 (0.57, 8.20)	0.261
Hispanic	3.12 (0.65, 14.89)	0.154
Comorbidities		
HTN	1.19 (0.56, 2.57)	0.649
Smoking	1.19 (0.59, 2.38)	0.628
Renal disease	0.99 (0.38, 2.58)	0.977
Aortic presentation		
Aortic aneurysm	0.77 (0.39, 1.53)	0.452
Aortic dissection	1.25 (0.58, 2.73)	0.571
Operative urgency		
Urgent/emergent	3.16 (1.44, 6.93)	0.004
Operative variables		
CPB time	1.02 (1.01, 1.03)	<0.001
Cross-clamp time	0.99 (0.98, 1.00)	0.005
HCA time	1.02 (0.99, 1.04)	0.189
Nadir bladder temperature	1.22 (1.06, 1.41)	0.006

Notes: Independent variables included race and the socioeconomic vulnerability index (SVI ≥ 75%). Confounders included age, body mass index (BMI), baseline systolic and diastolic blood pressure, comorbidities (hypertension, smoking, renal disease), aortic presentation (aneurysm, dissection), operative urgency (urgent/emergent), and operative variables (cardiopulmonary bypass [CPB] time, cross-clamp time, hypothermic circulatory arrest [HCA] time, and nadir bladder temperature). Statistically significant variables included urgent or emergent operative status (OR: 3.16, 95% CI: 1.44–6.93, *p* = 0.004), CPB time (OR: 1.02, 95% CI: 1.01–1.03, *p* < 0.001), cross-clamp time (OR: 0.99, 95% CI: 0.98–1.00, *p* = 0.005), and nadir bladder temperature (OR: 1.22, 95% CI: 1.06–1.41, *p* = 0.006). Age and BMI demonstrated borderline significance.

Postoperative readmission analysis revealed a complex interplay of demographic and intraoperative factors ( Table 4 ). Lower BMI was inversely associated with readmission risk (OR: 0.97, 95% CI: 0.94–1.00, *p* = 0.025), whereas longer CPB times were linked to higher readmission rates (OR: 1.01, 95% CI: 1.00–1.01, *p* = 0.024). Additionally, race demonstrated significant associations, with African American (OR: 0.24, 95% CI: 0.06–0.96, *p* = 0.043) and Caucasian (OR: 0.55, 95% CI: 0.31–0.97, *p* = 0.039) populations experiencing lower odds of readmission compared with other groups. Notably, the modest protective effect of prolonged cross-clamp time (OR: 0.99, 95% CI: 0.99–1.00, *p* = 0.034) warrants further investigation to clarify its clinical implications. Together, these findings highlight the importance of demographic considerations and tailored intraoperative strategies in reducing readmission risks, supporting a personalized approach to postoperative care.

**Table 4:** Multivariate logistic regression analysis evaluating predictors of postoperative readmission following aortic arch surgery

Variable	OR (95% CI)	*p* -Value
Patient characteristics		
Age	1.00 (0.99, 1.02)	0.634
BMI	0.97 (0.94, 1.00)	0.025
Baseline systolic BP	1.00 (0.99, 1.01)	0.729
Baseline diastolic BP	0.99 (0.97, 1.00)	0.091
SVI ≥ 75%	1.18 (0.75, 1.85)	0.487
Race		
African American	0.24 (0.06, 0.96)	0.043
Asian	0.26 (0.07, 1.01)	0.051
Caucasian	0.55 (0.31, 0.97)	0.039
Hispanic	0.73 (0.34, 1.58)	0.429
Comorbidities		
HTN	1.34 (0.90, 2.00)	0.155
Smoking	0.95 (0.63, 1.41)	0.788
Renal disease	1.51 (0.90, 2.55)	0.120
Aortic presentation		
Aortic aneurysm	0.82 (0.53, 1.28)	0.387
Aortic dissection	1.21 (0.75, 1.95)	0.441
Operative urgency		
Urgent/emergent	0.92 (0.58, 1.46)	0.721
Operative variables		
CPB time	1.01 (1.00, 1.01)	0.024
Cross-clamp time	1.00 (0.99, 1.00)	0.074
HCA time	1.00 (0.98, 1.02)	0.965
Nadir bladder temperature	1.06 (0.97, 1.16)	0.176

Notes: Independent variables included race and the socioeconomic vulnerability index (SVI ≥ 75%). Confounders included age, body mass index (BMI), baseline systolic and diastolic blood pressure, comorbidities (hypertension, smoking, renal disease), aortic presentation (aneurysm, dissection), operative urgency (urgent/emergent), and operative variables (cardiopulmonary bypass [CPB] time, cross-clamp time, hypothermic circulatory arrest [HCA] time, and nadir bladder temperature). The table presents odds ratios (OR) with 95% confidence intervals (CI) and *p* -values. Significant predictors of postoperative readmission included lower BMI (OR: 0.97, 95% CI: 0.94–1.00, *p* = 0.025), African American race (OR: 0.24, 95% CI: 0.06–0.96, *p* = 0.043), Caucasian race (OR: 0.55, 95% CI: 0.31–0.97, *p* = 0.039), and longer CPB time (OR: 1.01, 95% CI: 1.00–1.01, *p* = 0.024).

## Discussion

The results of the study demonstrate a clear lack of access to care for underrepresented groups. The demographics of the patient cohort are not consistent with the surrounding city's demographics, with an underrepresentation of non-Caucasian ethnicities. Ethnicity alone plays a statistically significant role in patient presentation, intraoperative characteristics, and postoperative ED utilization. For example, African Americans present with a higher baseline mean arterial pressure (MAP), present urgent or emergently, and are more likely to present with dissection pathology. African American and Hispanic individuals present at a younger age, and African Americans are also more likely to have procedure-related ED presentations within 1 year of surgery, as compared with other ethnicities. However, when investigating ethnicity alone, no statistically significant differences are seen for in-hospital morbidity, in-hospital mortality, readmission, or follow-up rates with cardiovascular providers.

Analysis based on SVI alone demonstrates that high SVI patients are vulnerable regardless of ethnicity. Regarding patient presentation, high SVI patients are more likely to present younger, present urgently or emergently, and have a history of smoking. Furthermore, they are more likely to present with more extensive pathology requiring a total arch replacement with longer operative times. Postoperatively, they more frequently require mechanical circulatory support and utilize the ED more frequently postdischarge. However, there is otherwise no significant difference in other morbidity or mortality for high SVI patients.

The data further demonstrate that several of the differences between ethnicities become statistically significant when the cumulative impact of SVI and ethnicity is considered, particularly as it relates to patient presentation, circulatory arrest time, and ED utilization postdischarge. This clearly establishes the importance of investigating the combined effect of different demographic variables, as analyzing these variables individually can hide important findings.

The results of this study were consistent with the findings of several prior studies. The finding that non-Caucasian patients present at a younger age, more urgently or emergently with a higher comorbidity burden has been demonstrated in previous research. 2 10 The current study supports those findings and indicates that specifically African American and Hispanic individuals present at a younger age, regardless of SVI. Our findings further support that high SVI patients are more likely to present younger, present urgently or emergently, and have a history of smoking. 2 11 The current study also supports that African American patients present more urgently or emergently and more frequently present with dissection pathology, regardless of SVI. A new finding from this study demonstrates that African American and Asian patients of all SVIs present with a higher baseline MAP. This study also demonstrated that high SVI across all ethnicities is associated with increased urgent and emergent presentation. The data further indicate that Asian patients with high SVI also present with dissection pathology at a higher rate than other ethnicities. These differences at presentation highlight the lack of access to care for certain ethnicities and high SVI groups. Interventions that improve access to preventative care and increase screening for aortic disease should be implemented and may have substantial positive impact.

Prior studies have shown that African American individuals were more likely to have more complex procedures with longer intraoperative times and longer circulatory arrest times. 10 However, this study shows that this difference is only statistically significant at high SVI. Our data demonstrate that when analyzing SVI alone, patients with high SVI are more likely to require total arch replacement leading to longer operative times.

Consistent with prior data on other proximal aortic surgery, this study demonstrates that SVI and ethnicity do not lead to significant difference for in-house morbidity and mortality postoperatively. 2 10 11 To further that, this study was the first to investigate follow-up rates with cardiovascular providers and rates of readmission, finding no significant differences. The lack of significant differences in in-hospital morbidity, mortality, readmission, and follow-up also demonstrates that once patients present to the hospital, they are receiving equitable care. The study further indicates that regardless of SVI, ED utilization 1 year postoperatively is higher for African Americans populations, but it does not lead to increased readmission rates. Overall, patients with high SVI alone also utilize the ED more frequently in the 1-year postoperative period. The increased ED utilization for these groups points to inequities in access to care that force these individuals to present directly to the ED for concerns not requiring readmission.

Our multivariate logistic regression analysis underscores the critical role of operative urgency and intraoperative variables in shaping postoperative outcomes. Urgent or emergent operative status significantly increased mortality risk, emphasizing the importance of preoperative optimization and judicious patient selection in these high-risk scenarios. Intraoperative factors also influenced readmission rates, with longer CPB times associated with higher readmission risk. These findings suggest that efforts to minimize bypass duration could mitigate postoperative complications and improve recovery trajectories.

While race and SVI were not independent predictors of mortality or readmission, this does not diminish the broader systemic and socioeconomic disparities highlighted in prior research. These results instead reinforce the importance of focusing on modifiable surgical and perioperative factors to improve outcomes universally, regardless of demographic or socioeconomic differences. Future studies should explore additional endpoints and potential confounders to better understand these complex relationships in diverse populations.

Several limitations to the study need to be acknowledged. First, while the study had a large overall sample size, specific cohorts within the dataset, such as the number of Asian patients, still have a small sample size of 20. Also, this is still a single-center study and thus subject to intrinsic limitations. The specific surgical techniques at this institution, and the access to care in and around the hospital and state, may limit the generalizability of the results. Furthermore, while all steps were taken to ensure thorough data collection for all cardiovascular provider follow-up visits, ED visits as well as unplanned readmissions within 1 year of surgery, including searching CareEverywhere, given that not all institutions participate in CareEverywhere it is possible that their frequency of visits and readmissions is underestimated. Additionally, SVI is calculated based on street address and five-digit zip code and is limited to neighborhood-level social vulnerability. It inherently generalizes the heterogeneity within that region as it is unable to factor in individual patient considerations. Given that SVI is calculated based on the census tract of an individual, it only provides the social vulnerability of a patient at a specific point in time and cannot capture any real-time changes. Despite its limitations, the use of neighborhood-level vulnerability indices, such as SVI, is a validated methodology frequently used in studies investigating health care disparities. 4 5 6 7 8 Another limitation of the study is the 11-year duration. Demographic and socioeconomic changes likely occurred in each ZIP code over that period, but since the SVI reflects the vulnerability of the neighborhood at the time the study was performed, it may not truly reflect the social disparities at the time of surgery for the earlier data. Lastly, it is important to consider that the study only captures patients who presented to the institution for an intervention. It is unable to account for access disparities that resulted in patient death or inoperable pathology.

Despite the limitations, this study emphasizes the importance of equitable access to health care for all patients undergoing aortic arch surgery. It demonstrates the importance of considering the cumulative effect of socioeconomic status and ethnicity, given analyzing them as individual variables often hides significance. The data provide insight into how social vulnerability affects different ethnicities and will help develop more targeted approaches to improving access to care. Specifically, it highlights the lack of access to preventative care faced by certain ethnicities and high SVI populations, given the significant differences in initial presentation. The study also sheds light on groups of individuals who may be at increased risk of certain pathologies at a younger age, necessitating early, targeted screenings. Furthermore, the data compel us to contemplate which interventions may improve postoperative access to care, so certain populations will not be forced to utilize the ED as their primary access point for health care. Further investigation is needed to determine specific barriers to equitable access to care, and what interventions, if any, have worked to mitigate these inequities.

## Conclusion

Clear lack of access to care exists for underrepresented groups as demonstrated by a patient population not reflective of city demographics, higher surgical acuity in socially vulnerable patients, and trends in ED usage after discharge. Furthermore, the ethnicity-only dataset hid significant differences within ethnicities between normal and high SVI groups, emphasizing the importance of considering the cumulative impact of both SVI and ethnicity. In contrast, patients belonging to minority groups, such as African American patients, also had apparent differences in presentation regardless of SVI, warranting further investigation to determine cause. Most importantly, approaches to expanding care need to be racially sensitive, applicable at all levels of care, and targeted toward high SVI groups.
